# Cerebral Small Vessel Disease and Renal Function: Systematic Review and Meta-Analysis

**DOI:** 10.1159/000369777

**Published:** 2014-12-24

**Authors:** Stephen D.J. Makin, F.A.B. Cook, Martin S. Dennis, Joanna M. Wardlaw

**Affiliations:** ^a^Clinical Research Fellow, Neuroimaging Sciences, University of Edinburgh, Edinburgh, UK; ^b^Division of Neuroimaging Sciences, University of Edinburgh, Bramwell Dott Building, Western General Hospital, Edinburgh, UK

**Keywords:** Lacunar stroke, Renal function, Small vessel disease, Proteinuria

## Abstract

**Background:**

The small vessel disease (SVD) that appears in the brain may be part of a multisystem disorder affecting other vascular beds such as the kidney and retina. Because renal failure is associated with both stroke and white matter hyperintensities we hypothesised that small vessel (lacunar) stroke would be more strongly associated with renal failure than cortical stroke. Therefore, we performed a systematic review and meta-analysis to establish first if lacunar stroke was associated with the renal function, and second, if cerebral small vessel disease seen on the MRI of patients without stroke was more common in patients with renal failure.

**Methods:**

We searched Medline and EMBASE for studies in adults with cerebral SVD (lacunar stroke or white matter hyper intensities (WMH) on Magnetic Resonance Imaging (MRI)), in which renal function was assessed (estimated glomerular filtration rate (eGFR) or proteinuria). We extracted data on SVD diagnosis, renal function, demographics and comorbidities. We performed two meta-analyses: first, we calculated the odds of renal impairment in lacunar (small vessel) ischaemic stroke compared to other ischaemic stroke subtypes (non-small vessel disease); and second, we calculated the odds of renal impairment in non-stroke individuals with WMH on MRI compared to individuals without WMH. We then performed a sensitivity analysis by excluding studies with certain characteristics and repeating the meta-analysis calculation.

**Results:**

After screening 11,001 potentially suitable titles, we included 37 papers reporting 32 studies of 20,379 subjects: 15 of stroke patients and 17 of SVD features in non-stroke patients. To diagnose lacunar stroke, 13/15 of the studies used risk factor-based classification (none used diffusion-weighted MRI). 394/1,119 (35%) of patients with lacunar stroke had renal impairment compared with 1,443/4,217 (34%) of patients with non-lacunar stroke, OR 0.88, (95% CI 0.6-1.30). In individuals without stroke the presence of SVD was associated with an increased risk of renal impairment (whether proteinuria or reduced eGFR) OR 2.33 (95% CI 1.80-3.01), when compared to those without SVD. After adjustment for age and hypertension, 15/21 studies still reported a significant association between renal impairment and SVD.

**Conclusion:**

We found no specific association between renal impairment and lacunar stroke, but we did find that in individuals who had not had a stroke, having more SVD features on imaging was associated with a worse renal function, which remained significant after controlling for hypertension. However, this finding does not exclude a powerful co-associate effect of age or vascular risk factor exposure. Future research should subtype lacunar stroke sensitively and control for major risk factors.

## Introduction

Impaired kidney function is associated with an increased risk of stroke [1], and reduced estimated glomerular filtration rate (eGFR) is associated with an increased risk of cerebral small vessel disease (SVD) such as white matter hyperintensities (WMH) [2], and lacunes. It has been proposed that SVD is a manifestation of an underlying multi-system endothelial disorder affecting the small vessels of the kidney, brain, heart, and retina [3], possibly mediated through inflammation [4,5].

All studies of renal function and SVD are faced with the challenge of disentangling whether these disorders are a common consequence from shared risk factors or represent a causative relationship. One way of doing this is to compare patients with different stroke subtypes, that is, to compare patients with lacunar (small vessel) stroke to patients with other stroke subtypes, or to compare the prevalence of renal disease in individuals with imaging-determined SVD features. We hypothesised that: patients presenting with a symptomatic recent ischaemic lacunar stroke may have a greater risk of renal impairment than patients with the other ischaemic stroke subtypes that are associated with embolism or large vessel disease. Additionally, individuals without symptomatic stroke may be at increased risk of SVD on imaging if they have renal impairment, after risk factor adjustment.

We performed a systematic review of the literature to establish first the risk of renal impairment in patients with lacunar stroke compared to patients in other ischaemic stroke subtypes, and second, the risk of renal impairment in non-stroke participants with SVD features on imaging (e.g. WMH, lacunes) compared to those without SVD.

## Methods

We followed the ‘Preferred Reporting Items for Systematic Reviews and Meta-Analyses' (PRISMA) guidelines [6]. We searched MEDLINE (1966-present) and EMBASE (1981-present) using OVID (version OvidSP_UI03.08.01.105), using the search terms in the Supplementary Information – last search was conducted on April 2013. SM and FABC independently reviewed the titles to identify the relevant papers and extracted the data. We resolved the disagreements through mutual discussion and consultation with JMW. We hand-searched the past editions of the journal ‘Stroke', and the abstracts of presentations at European Stroke Conferences from 2006-2013 (published in Cerebrovascular Diseases), and the reference lists of relevant review papers. We deemed a paper to be potentially relevant if it included a reference to the measurement of renal function or mentioned SVD in adult humans in the title, and then went on to read the whole paper.

We included studies that measured the renal function in living humans with either symptomatic lacunar stroke or imaging features of SVD. We included studies that described WMH, lacunes, or ‘silent cerebral infarcts', as these terms are commonly used to refer to SVD on imaging [7,8], but excluded atrophy as this was inconsistently reported. We excluded studies that were performed on animals or post mortem, that measured renal disease but did not investigate the renal function (e.g. studies of renal biopsy findings), and studies that only included participants with renal impairment.

We extracted data on study population; location (community or hospital); inclusion and exclusion criteria; diagnosis of stroke subtype; the details of any imaging and image analysis methods; the definition used of SVD; how renal function was measured and defined; blinding; the differences in risk factors between the participants with and without stroke/SVD; the numbers of participants with and without stroke/imaging features of SVD who had renal impairment; and any adjusted or unadjusted summary statistics such as Odds Ratios (OR). We contacted the authors if it was apparent that the data had been acquired but was not reported in the paper.

We considered renal impairment to be either a reduced eGFR (<60 ml/min, Stage 3 Chronic Kidney Disease) or albuminuria – either micro (30-300 ml/l) or macro (>300 ml/l). For the purpose of the meta-analysis, we used the definitions of renal impairment, lacunar stroke, and SVD from the individual studies within our overall definition.

We carried out two meta-analyses. First, we compared the risk of renal impairment in patients with lacunar stroke to patients in other ischaemic stroke sub-groups. Second, we compared the risk of renal impairment in non-stroke participants with SVD on imaging to those without SVD. The meta-analysis included all the studies in that had dichotomised participants into those with and without renal impairment. If a study gave the mean and standard deviation (SD) of the eGFR, we assumed a normal distribution and calculated the number of patients with an eGFR below 60. We used a random effects model to account for differences in underlying study methodology. We performed analyses using Stats Direct (StatsDirect statistical software version 2.7.9 http://www.stats​direct.com. England: StatsDirect Ltd., 2008) and RevMan (Version 5, Cochrane Collaboration).

Each meta-analysis followed the same procedure. We calculated the summary OR of all studies using the Mantel-Haenszel random effects model and assessed heterogeneity using the I^2^ statistic. We used a funnel plot to examine for publication bias. To assess the causes of heterogeneity and the risk of bias in individual studies we performed a sensitivity analysis by excluding certain studies with various characteristics and repeating the meta-analysis. Lastly, we examined the summary statistics of the individual studies that had carried out a multivariable analysis accounting for age and hypertension. No protocol was published externally.

## Results

**Fig. 1:**
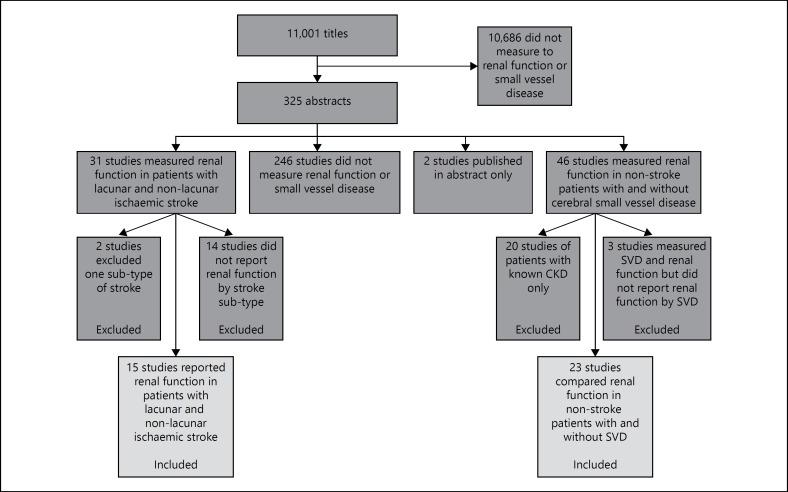
Flow chart of search results.

We identified 11,001 potentially suitable titles (fig. 1). Of these, we excluded 1,0676 titles because they did not refer to either renal function, or SVD in adults, and read the abstract or full paper of the remaining 325 references. We excluded 246 studies because they did not measure both renal function and SVD, two because they were published only in abstract, 20 because they only included patients with established kidney disease, 14 because they did not report the renal function of patients by stroke subtype and a further two because they only included one particular stroke sub-type. Full details of excluded studies are available on request.

We included 38 papers describing 32 studies of 20,379 participants: 15 studies [9,10,11,12,13,14,15,16,17,18,19,20,21,22,23] (45 of studies, 37% of patients) of stroke patients, and 17 studies [24,25,26,27,28,29,30,31,32,33,34,35,36,37,38,39,40,41,42] of healthy volunteers or non-stroke patients who had SVD defined on imaging alone (55 of studies, 63% of participants).

### Critical Appraisal of 15 Studies of Stroke Patients

Study characteristics are summarised in tables 1 and 2. Thirteen studies [9,10,13,14,15,16,17,18,19,21,22,23,43] (87 of studies of stroke patients, 88% of stroke patients) recorded stroke subtype (lacunar or non-lacunar), while three recorded renal function in stroke patients with and without WMH on Magnetic Resonance Imaging (MRI) [9,11,12].

Most studies were from developed countries and varied with respect to inpatient and outpatient recruitment. One study [20] (n = 958) included only young patients with a stroke but the rest included all ages (overall the mean age was 67). All studies measured the renal function as soon as possible after stroke, with the exception of one study of 96 patients which assessed renal function 6-8 weeks post-stroke [14].

### Characteristics of the 13 Studies that Subtyped Stroke

Of the 13 studies that subtyped the stroke, only one [16] used the Oxfordshire Community Stroke Project (OCSP) [44] classification (8 of studies, 7% of sub-typed stroke patients); all other studies used the risk-factor based Trial of Org 10,172 in Acute Stroke Treatment (TOAST) classification [45]. No studies used diffusion weighted MRI (DWI-MRI) in the acute phase. Four studies measured proteinuria [13,14,19,22] (31 of studies, 9% of sub-typed stroke patients), and nine [9,15,16,17,18,20,21,22,23] measured eGFR. No studies reported whether there were any differences in vascular risk factors between patients with lacunar and non-lacunar stroke.

### Characteristics of 17 Studies of Non-Stroke Patients

Seventeen studies [24,25,26,27,28,29,30,31,32,33,34,35,36,37,38,39,40,41,42,46,47] of 13,164 participants (mean age 65) measured renal function and MRI features of SVD in participants without a symptomatic stroke (tables 1 and 2). Eight studies [26,30,31,32,33,34,35,39] were of healthy volunteers; four were of diabetic patients (10% of subjects); two [28,29] were of patients with any of a variety of vascular risk factors, one was of hypertensive patients alone [36], and another included both hypertensive patients and their siblings (12% of subjects) [34,37].

Ten studies [24,25,26,28,29,32,34,36,38,39] reported the blood pressure of participants with and without SVD (either as the percent of subjects previously diagnosed with hypertension or as the mean systolic blood pressure on examination), and two studies [24,36] reported little difference between the participants with and without SVD (a difference of less than 2% in the proportion of participants or a difference of less than 5 mm Hg in systolic blood pressure between groups).

Ten studies [26,28,29,31,32,34,37,38,39] excluded participants with previous stroke, nine [24,25,26,28,31,33,38,39,42] excluded those with the most severe renal impairment, and all measured the MRI features of SVD.

Five studies [24,28,35,36,38,40] used a method such as the Fazekas score [48] to grade WMH, six [25,26,29,32,34,38] counted the number of silent brain infarcts, and five [27,30,31,37,42] used an automated measure to quantify the volume of WMH. Nine studies [24,26,27,28,29,33,34,36,41,42] (46% of participants) were read by an observer blinded to the clinical details. Eight studies [26,27,30,31,34,35,36,37] (70% of non-stroke participants) measured eGFR, six [24,25,33,38,39,42] measured proteinuria, three [28,32,40] measured both, and one study [29] measured serum creatinine. Thirteen studies [24,25,26,27,28,29,30,31,32,34,35,36,39,42] carried out a multivariate analysis accounting for (at least) age and hypertension. Twelve papers treated SVD as a binary variable (i.e., present or absent) and performed binary logistic regression, while seven treated it as a continuous variable and used linear regression.

### Meta-Analysis of the Risk of Renal Impairment in Lacunar Stroke Versus Other Stroke Subtypes

First, we performed a meta-analysis of the studies reporting the numbers of lacunar and non-lacunar stroke patients with renal impairment (defined as proteinuria or an eGFR below 60 ml/min): 12 studies of 5,338 patients [9,10,13,14,15,16,17,18,20,21,22,23]. We excluded a study of patients who suffered deterioration in renal function after stroke [19] and two studies of stroke patients that measured WMH volume, not stroke subtype [11,12.]

**Fig. 2:**
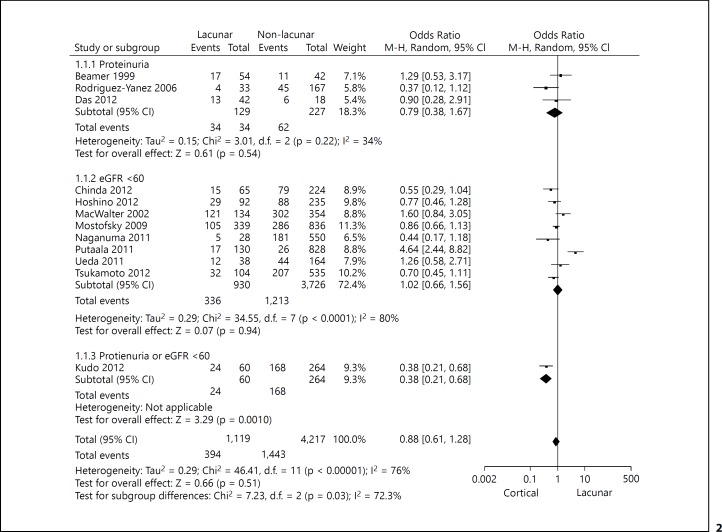
A forest plot demonstrating the results of the meta-analysis of studies of renal function in patients with lacunar and cortical stroke.

Overall there was no specific association between renal function and stroke subtype – lacunar versus non-lacunar: 394/1,119 (35%) of patients with lacunar stroke had renal impairment, compared with 1,443/4,217 (34%) of patients with non-lacunar stroke (fig. 2) OR 0.88, 95% confidence interval (CI) 0.61-1.28. There was a high degree of heterogeneity (inconsistency) with an I^2^ of 76%. When comparing patients with lacunar and non-lacunar stroke there was no statistically significant difference in the odds of proteinuria, OR 0.79 (95% CI 0.38-1.67), or eGFR <60, OR 1.02 (95% CI: 0.66-1.56). No studies of stroke patients performed a multivariable analysis accounting for risk factors. Funnel plots (online suppl. fig. A, B; see www.karger.com/doi/10.1159/000​369777) did not indicate publication bias.

**Fig. 3:**
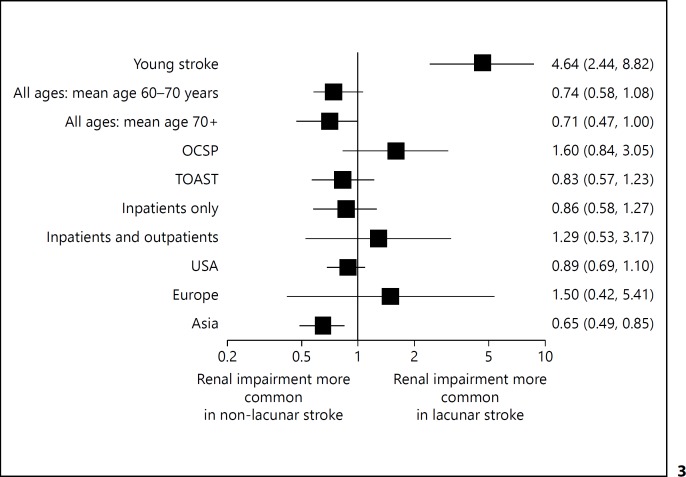
The Odds Ratio of renal impairment in patients with lacunar stroke compared to other stroke sub-type for different subgroups of studies.

Sub-group analysis (fig. 3) suggested an association between lacunar stroke and impaired renal function in younger patients: a study that only included participants aged 15-49 [20] found a fourfold risk of renal impairment when compared to other subtypes, OR 4.64 (95% CI: 2.44-8.82); whereas in studies of patients with a mean age of 70 or greater there was no significant difference between subtypes. In Asian studies (Japan [9,15,17,21,22,23], and Bangladesh [10]) patients with lacunar stroke had a reduced risk of renal impairment OR 0.65 (95% CI 0.49-0.85). Neither the method of stroke sub-typing (OCSP or TOAST), nor a study that recruited only inpatients, affected the lack of association between lacunar stroke subtype and renal impairment.

### Meta-Analysis of the Risk of Renal Impairment in Non-Stroke Participants

We included 12 studies [24,25,26,32,34,35,36,38,39,40,42,46] of 11,269 participants in this analysis. We excluded studies that only measured Cystatin C [41] or serum creatinine [29]; those that used microalbuminuria as the dependent variable [33]; or those that did not dichotomise renal impairment or SVD [30,31,37,47]. For the two studies [35,36] that did not report a total WMH score but instead reported the findings for deep and periventricular WMH separately, we included the data for periventricular lesions in the meta-analysis, as these are more prevalent. Two studies reported the results of eGFR and proteinuria in separate papers [32,39,40,49]; therefore, we ensured that each participant only contributed once to each calculation.

### Unadjusted Odds of Renal Impairment in Participants with and without Imaging Features of Small Vessel Disease

**Fig. 4:**
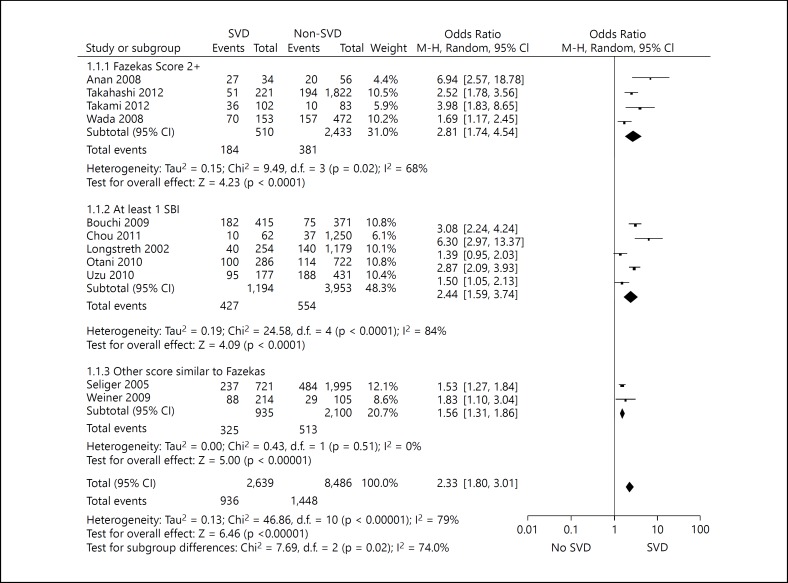
Meta-analysis of the risk of renal impairment in non-stroke patients with SVD compared to those without SVD.

The OR of renal impairment (either eGFR or proteinuria) in participants with SVD compared to those without was 2.33 (95% CI 1.80-3.01), with an I^2^ of 78.2% (fig. 4). Studies that recorded ‘silent brain infarcts' had a higher degree of heterogeneity (I^2^ 84%), which may represent the range of different lesions described as ‘silent brain infarcts'. Further meta-analysis of the unadjusted data revealed that participants with SVD were twice as likely to have proteinuria compared with participants without SVD: OR 2.00 (95% CI 1.44-2.78) with a moderate degree of heterogeneity (I^2^ 54.1%); and almost three times as likely to have an eGFR <60, OR 2.82 (95% CI 1.94-4.10), but with a high degree of heterogeneity: I^2^ 84.4%.

**Fig. 5:**
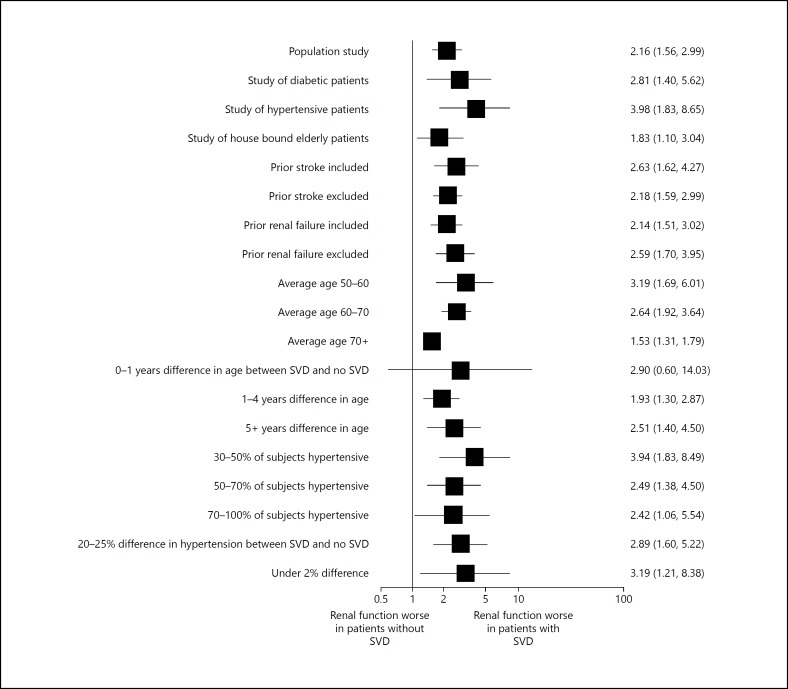
The Odds Ratio of renal impairment in non-stroke patients with small vessel disease compared to those without for different subgroups of studies.

We investigated whether the relationship between SVD and renal function varied in studies of particular groups of participants (fig. 5). For studies of younger patients (average age 50-60) there was a stronger relationship between impaired renal function and SVD (OR 3.19, 95% CI 1.69-6.01) in comparison with studies of patients over 70 (OR 1.53, 95% CI 1.53-1.79). Other study factors had little consistent effect on the relationship between renal function and SVD. A funnel plot (online suppl. fig. B) revealed little evidence of publication bias.

### Risk Factor Adjusted Odds of Renal Impairment in Participants with and without Imaging Features of Small Vessel Disease

Nineteen studies [24,25,26,27,28,29,30,31,32,34,35,36,37,39,40,42,46,47,49] calculated odds ratios adjusted for age, hypertension and a variety of other risk factors; but we could not carry out a meta-analysis of the adjusted ORs as all studies adjusted for slightly different parameters.

After adjusting for (at least) age and hypertension, nine studies [24,25,26,27,31,35,42,46,47] reported a significant association between renal function and SVD, but with a smaller OR than the unadjusted statistic. However, three studies [28,34,37] reported no significant link between renal function and SVD after adjustment for age and hypertension; one [49] found a significant link between proteinuria and SVD, but not between eGFR and SVD; one [30] found a significant link between renal impairment for participants with periventricular, but not deep WMH, whereas another [36] found that there was a significant link between renal impairment and deep (but not periventricular) WMH.

## Discussion

Participants with cerebral SVD features on imaging were found to be at increased risk of renal impairment compared to participants without SVD, but patients with a symptomatic lacunar stroke were at no more risk of renal impairment than patients with a non-lacunar stroke.

An association between lacunar sub-type and renal impairment could have been missed by the studies of stroke patients because the sub-typing (largely based on clinical and CT diagnosis) was not sufficiently precise to distinguish lacunar from non-lacunar stroke [50]. No studies used a gold-standard means of stroke sub-typing, namely risk-factor free clinical sub-typing aided by DWI-MRI in the acute phase. As some studies [51] have suggested that lacunar stroke affects patients at a younger age than non-lacunar stroke, the lack of adjustment for age in the analysis of patients with symptomatic stroke may have masked an association between lacunar stroke subtype and impaired renal function. In a study of younger patients [20] there was a stronger association between impaired renal function and lacunar stroke than with other stroke subtypes, which is interesting as an association between renal function and lacunar stroke may not be present across all age groups as it could be diluted by older patients having more heterogeneous risk factors.

We did not investigate whether the different non-lacunar sub-types were associated with renal impairment as we were investigating the association between small vessel disease and renal impairment.

There was a high level of heterogeneity throughout the literature with different methods of measuring SVD, stroke sub-type, proteinuria, and eGFR. No studies reported differences in risk factors between patients with and without lacunar stroke, which limited our investigation of covariates such as hypertension. Almost all studies measured renal impairment in the acute phase, leading to potential confounding by dehydration, which is common after stroke [52].

This work has been hampered by the lack of a standardised definition of SVD, with various studies using definitions such as silent brain infarcts, and ‘lacunes' to represent similar imaging findings. This problem was compounded by various definitions of proteinuria/albuminuria. Future studies should use the recently published standardised imaging definitions of SVD [7].

The strengths of this review include a comprehensive literature search incorporating studies from America, Europe and Asia with no language exclusions. In addition to the relationship between reduced eGFR and silent lesions investigated by Vogels et al. [2] we have included patients with proteinuria, symptomatic stroke, and a meta-analysis. Weaknesses comprise the inclusion of only dichotomised studies in the meta-analysis: some more recent studies investigated the continuous relationship between WMH volume and renal impairment. We were not able to fully investigate the effects of powerful confounding factors (e.g. age and hypertension), because it was not possible to carry out a meta-analysis of the adjusted ORs from multivariate analysis as they had all corrected for different confounders.

The apparently strong link between ‘silent' SVD and renal impairment in studies of stroke-free patients was not seen in studies of symptomatic stroke. This calls into question the hypothesis that cerebral and renal SVD are directly associated as part of the same multi-system disease rather than representing end organ damage from shared risk factors particularly of hypertension. However, it is difficult to draw firm conclusions due to a high degree of heterogeneity and imprecise stroke sub-typing.

Studies of non-stroke participants should use age-matched controls and carry out multivariate analysis of confounding factors. As over 10,000 participants have already undergone MRI and the measurement of renal function, it should be possible to achieve this by re-analysis of the existing data using a well-resourced individual patient data meta-analysis. Future studies of stroke patients should perform careful sub-typing using risk factor-free clinical classification (i.e. OCSP [44] aided by early DWI-MRI), measure proteinuria and eGFR outside the acute phase, and compare with age-matched non-lacunar stroke controls accounting for variations in risk factors.

## Funding

S.D.J.M. is supported by a Wellcome Trust Project Grant (WT088134/Z/09/A). J.M.W. is supported by the Scottish Funding Council through the Scottish Imaging Network, A Platform for Scientific Excellence (SINAPSE) Initiative (http://www.sinapse.ac.uk). The study was independent of the funders.

## Disclosure Statement

The authors have no conflict of interest.

## Figures and Tables

**Table 1:** Characteristics of studies included in the systematic review

Study and population		Lacunar stroke definition	Imaging	Definition of renal impairment	Lacunar ischaemic stroke impaired/total	Non-lacunar ischaemic stroke impaired/total
Studies which compared renal function between lacunar and non-lacunar stroke						

Beamer [14], 1999 USA	Setting: 2 hospitals	TOAST	not clear	Proteinuria >20 mg/l	17/54	11/42
	Included: 96 patients up to 7 days post-stroke					
	Excluded: UTI and dialysis					

Das [10], 2012 Bangladesh	Setting: neurology department	TOAST	CT/MRI	proteinuria 20–200 ml/l	13/42	6/18
	Included: 60 patients up to 4 weeks post-stroke					
	Excluded: known CKD					

MacWalter [16], 2002 UK	Setting: teaching hospital	OCSP	CT	eGFR <66	121/134	302/354
	Included: 488 patients 48 h post stroke					
	Excluded: dialysis					

Rodríguez-Yáñez [13], 2006 Spain	Setting: teaching hospital	TOAST	CT	proteinuria <30 mg/l	4/33	45/167
	Included: 200 patients within 24 h					
	Excluded: TPA/trial drug, brainstem stroke or known renal disease					

Tsagalis [19], 2009 Greece	Setting: teaching hospital stroke data bank	TOAST	CT	>50% increase in creatinine from baseline	72/378	403/1454
	Included: 2,155 patients <48 h post stroke with 2× creatinine measurements					
	Excluded: previous stroke					

Naganuma [21], 2011 Japan	Setting: registry of thrombolysis patients in 10 stroke units	TOAST	CT/MRI	eGFR <60	5/28	181/550
	Included: 578 patients who were thrombolysed for ischaemic stroke					
	Excluded: patients disabled prior to stroke					

Mostofsky [18], 2009 USA	Setting: emergency department	TOAST	CT/MRI	eGFR <60	105/339	286/836
	Included: 1,175 consecutive patients					
	Excluded: IN-hospital stroke					

Ueda [9], 2011 Japan	Setting: stroke unit	TOAST	MRI	eGFR <60	12/38	44/164
	Included: 202 consecutive ischaemic stroke patients					
	Excluded: acute kidney injury					

Putaala [20], 2011 Finland	Setting: Helsinki young stroke registry	TOAST	not clear	eGFR <60	17/130	26/828
	Included: 958 first stroke patients age 15–19					
	Excluded: incomplete data or creatinine measured 30 days post-stroke					

Hoshino [17], 2012 Japan	Setting: Neurology Department	TOAST	CT	eGFR <60	29/92	88/235
	Included:475 stroke patients					
	Excluded: severe renal dysfunction, pre stroke disability					

Kudo [22], 2012 Japan	Setting: single hospital	TOAST	CT/MRI	eGFR <60 and/or24/60 proteinuria		168/264
	Included: 525 stroke patients					
	Excluded: missing data					

Tsukamoto [23], 2012 Japan	Setting: neurology department	TOAST	CT/MRI	eGFR <60	32/104	207/535
	Included: 639 consecutive stroke patients					
	Excluded: dialysis patients					

Chinda [15], 2012 Japan	Setting: single hospital	TOAST	CT/MRI	eGFR <60	15/65	79/224
	Included: 451 consecutive stroke patients					
	Excluded: presented later than 7 days after stroke					

Studies which compared renal function in stroke patients with WMH to those without WMH						

Oksala [11], 2010 Finland	Setting: single hospital	NA	1.0T MRI	eGFR <60	96/203	56/175
	Included: 378 consecutive ischaemic stroke patients aged 55–85					
	Excluded: patients who were not Finnish or not living in Helsinki					

Rost [12], 2010 USA	Setting: Emergency Department	NA	1.5T MRI	eGFR as a continuous relationship	low eGFR correlated with WMH volume, r = −0.003, p = 0.002	
	Included: 523 consecutive ischaemic stroke patients					
	Excluded: patients without a lesion on MRI					

**Study**	**Patients**	**Imaging**	**Definition SVD**	**Measure of renal function**	**Subjects with SVD impaired/total**	**Subjects without SVD impaired/total**

Studies of patients with MR imaging features of SVD, but no symptomatic stroke						

Uzu [38], 2010 Japan	Setting: diabetic outpatient clinic	1.5T MRI	1+ SBI definition not given	micro albuminuria (30–299 ml/1)	95/177^2^	188/431
	Included: 608 type 2 diabetic					
	Excluded: IHD, cancer, steroid use, heavy proteinuria 300 ml/l+, renal impairment					

Ikram [30], 2008 Netherlands	Setting: Rotterdam study: population based study of 7,983 participants over 50	1.5T MRI	automated measurement of WMH volume	eGFR	for each SD decrease in eGFR there was a significant increase in OR of WMH 0.16 (0.04–0.29)	
	Included: subgroup of 484 participants aged 60–90 stratified by sex and age					
	Excluded: patients with known dementia, or who could not have MRI					

de Bresser [27], 2010 Netherlands	Setting: patients aged 56–80 with diabetes recruited though their General Practitioners	1.5T MRI	automated measurement of WMH volume	albuminuria >0.03 g/1	baseline albuminuria was associated with a non-significant increase in WMH at 2 years	
	Included: 122 patients with Type 2 diabetes					
	Excluded: patients with psychiatric and neurological disorders, heavy alcohol use and dementia					

Seliger [34], 2005 USA 40	Setting: Cardiovascular Health Study 5,888 individuals over 65 selected randomly from medicare lists	not clear	1+ infarct-like lesion ≥3 mm in a patient without a history of stroke	eGFR <60	237/789	484/1,995^2^
	Included: 2,784 participants selected for MRI					
	Excluded: previous stroke and TIA					

Giele [29], 2004 Netherlands	Setting: second manifestations of ARTertial disease (SMART) study	1.5T MRI	1+ CSF filled lesion ≥3 mm	mild renal impairment: eGFR 80–50 Severe renal impairment eGFR <50	age adjusted OR for presence of silent infarcts in: mild renal impairment 1.6 (0.7–3.5)	
	Included: 308 patients with first presentation of atherosclerotic disease					
	Excluded: previous stroke or TIA				Severe renal impairment 7.3 (2.1–25.2)	

Wada [39], 2007; [40], 2008; [41], 2010 Japan	Setting: population study of all 61 and 72 year olds from two towns	0.3 & 0.5T MRI	Fazekas score of either 2 or 3 (not specified whether deep or periventricular)	presence of micro albuminuria: cut off not clear	95/177	188/431
	Included: 608 participants					
	Excluded: history of stroke, current UTI					
				
				eGFR <60 or urinary ACR <30	70/143	157/508
				
				Cystatin C	OR of moderate or severe WMH, per SD increase in cystine C 1.48 (1.22–1.78) (unadjusted)	

Weiner [42], 2009 USA	Setting: clients of a home care service for low income people over 60	1.5T MRI	a score of 2/10 or more on an unvalidated qualitative WMH rating scale	microalbuminuria (17 mg/g+ in men and 25 mg/g+ in women)	88/214	29/105
	Included: 319 participants					
	Excluded: participants who were unable to consent, non-English speakers, had a visual or hearing disability, on dialysis or unable to provide a urine specimen					

Otani [32], 2010 Japan	Setting: population study of one town	0.5T MRI	at least 1 hyperintensity on T2 between 3 and 15 mm	eGFR <60	100/286	186/722
	Included: 1,008 participants aged over 55					
	Excluded: previous stroke or TIA					

Bouchi [25], 2010 Japan	Setting: patients with type 2 diabetes who had an MRI for any reason at a single hospital	1.5T MRI	T2 hyperintensity ≥3 mm	eGFR <60	182/415	75/371
	Included: 786 participants					
	Excluded: patients with type 1 DM, pregnancy, infection, cancer, or eGFR under 15					

Chou [26], 2011 Taiwan	Setting: healthy volunteers from Taipai City	1.5T MRI	T2 hyperintensity ≥3 mm	eGFR 30–60	10/62	37/1,250
	Included: 1,312 participants					
	Excluded: previous stroke, current fever, eGFR <30					

Anan [24], 2008 Japan	Setting: outpatient endocrinology clinic	1.5T MRI	the presence of WMH with Fazekas score ≥2 – unclear if deep or periventricular	urinary albumin in the range of 30–299 mg/24 h	27/34	20/56
	Included: 90 patients with type 2 diabetes					
	Excluded: patients with IHD, macro-albuminuria, high creatinine, or insulin use					

Eguchi [28], 2004 Japan	Setting: asymptomatic patients having an annual health check	0.5T MRI	at least 1 hyperintensity on T2 between 3 and 15 mm	correlation between serum creatinine and no of WMH	serum creatinine correlated with number of WMH, r = 0.2, p < 0.006	
	Included: 170 patients aged 42–89 with 3 or more vascular risk factors					
	Excluded: renal or liver failure, secondary or malignant hypertension					

Khatri [31], 2007 USA	Setting: randomly selected residents of Manhattan	1.5T MRI	automated measurement of WMH volume	correlation between creatinine clearance and WMH volume	creatinine clearance of 15–60 ml/linked to log WMH volume (0.322; 95% CI, 0.095–0.550)	
	Included: 615 participants over 40 who had a telephone and could consent					
	Excluded: those with a history of stroke or eGFR <15					

Takahashi [35], 2012 Japan	Setting: asymptomatic patients presenting for a ‘brain check’.	1.5T MRI	score of ≥2 on Fazekas score – deep and periventricular lesions analysed separately	eGFR <60	deep WMH 89/465	deep WMH 156/1,571
	Included: 2,043 healthy volunteers.					
	Excluded: participants with a history of stroke, neurological, or heart diseases					
					
					periventricular WMH: 51/221	periventricular WMH: 194/1,822

Takami [36], 2012 Japan	Setting: outpatient hypertension clinic	1.5T MRI	deep WMH: cases if Fazekas score ≥2. Periventricular WMH cases if Fazekas score ≥1	eGFR <60	deep WMH 31/75	deep WMH 16/110^2^
	Included: 185 participants					
	Excluded: patients with AF					
					
					periventricular WMH 36/102	periventricular WMH 10/83^2^

Turner [37], 2011 USA	Setting: members of sibling pairs where one was hypertensive	1.5T MRI	automated measurement of WMH volume on FLAIR	correlation between serum creatinine and WMH volume	correlation between serum creatinine and WMH volume. Age adjusted correlation coefficient = 0.54	
	Included: 1,585 participants					
	Excluded: secondary hwypertension, known CKD and previous stroke					

Ravera [33], 2002 Italy	Setting: patients from one centre who were enrolled in a large study on complications of microalbuminuria in untreated patients with mild-moderate hypertension	1.5T MRI	a count of number of lacuanes: 3–15 mm lesion dark on T1, light on T2	no of lacunes in 11 patients with microalbuminuria against the number of lacunas in 11 patients without microalbuminuria 82 of patients with microalbuminuria had incident lacunes vs. 27% of patients without		
	Included: 22 patients with microalbuminuria, 22 controls without					
	Excluded: patients with cancer, liver disease, IHD, diabetes, obesity, and Dementia					

^1^All studies excluded patients unable to have MRI

2 Calculated from mean and SD assuming a normal distribution. UTI = Urinary tract infection; TOAST = trial of org 10,172 in acute stroke treatment; CKD = chronic kidney disease; CT = computerised topography; MRI = magnetic resonance imaging; OCSP = oxfordshire community stroke project; eGFR = estimated glomular filtration rate; TPA = tissue plasminogen antigen; WMH = white matter hyperintensities; SD = standard deviation; IHD = ischaemic heart disease; TIA = transient ischaemic attack; CSF = cerebrospinal fluid; OR = odds ratio; ACR = albumin creatinine ratio.

**Table 2:** Summary of characteristics of studies

Characteristic	Number of studies	% of studies	% of subjects
Stroke patients	14	45	37
Non stroke	17	55	63

Studies of stroke patients		% of studies of stroke patients	% of stroke patients

Recorded stroke sub-type	13	87	88
Inpatients and outpatients	1	7	1.2
Inpatients only	12	80	96
USA	3	20	22
Europe	5	33	48
Asia	7	47	29

Studies of subtyped stroke patients		% of studies that subtyped stroke	% of patients with subtyped stroke

OCSP	1	8	7
TOAST	12	92	93
CT Imaging	4	31	40
MRI	2	15	12
CT and MRI	4	31	17
Measured proteinuria	4	31	9
Measured eGFR	9	69	69
Patients that developed acute kidney injury	1	8	26

Studies of imaging features of SVD patients		% of studies of imaging features of SVD	% of subjects in the studies of imaging features of SVD

Healthy volunteers	8	47	57
Diabetic patients	4	24	10
Any vascular risk factor	2	12	4
Hypertensive patients	1	6	0.2
Hypertensive patients and their siblings	1	6	11
Excluded previous stroke	10	59	84
Excluded severe renal impairment	9	53	35
1.5T	14	82	86
0.5T	3	18	14
Fazekas or similar	5	29	24
Count of silent brain infarcts	6	35	52
Automated measure of the volume of WMH	5	29	24
Images analysed by a blinded observer	9	53	46
Measured eGFR	8	47	70
Measured proteinuria	5	29	13
Measured both	3	18	14
Measured serum creatinine	1	6	2
Multivariate analysis	13	76	81
